# Bleeding and hemostatic defects in Lowe syndrome: the role of OCRL in platelets

**DOI:** 10.3389/fcell.2026.1886446

**Published:** 2026-07-02

**Authors:** Ana Bura, Antonija Jurak Begonja

**Affiliations:** Faculty of Biotechnology and Drug Development, University of Rijeka, Rijeka, Croatia

**Keywords:** bleeding, Lowe syndrome, megakaryocytes, ocrl, platelets

## Abstract

Lowe syndrome (LS) is an X-linked disorder caused by OCRL mutations, encoding a phosphatidylinositol 5-phosphatase. OCRL regulates phosphatidylinositol-(4,5)-bisphosphate [PI(4,5)P_2_]-dependent cytoskeletal dynamics and membrane trafficking in multiple cell types, including platelets. Clinically, LS manifests with cataracts, hypotonia, developmental delay, and progressive kidney disease. A subset of LS individuals shows bleeding tendency and occasional mild thrombocytopenia despite normal coagulation tests. Studies reveal impaired thrombus growth under flow, delayed platelet spreading on fibrinogen, abnormal clot retraction, and altered platelet formation with circulating barbell-shaped immature platelets, supporting a platelet dysfunction phenotype in LS, though exact molecular mechanisms remain undefined. Experimental studies suggest OCRL controls PI(4,5)P_2_ pools required for coordinated actin and microtubule remodeling during platelet adhesion and thrombopoiesis. Pharmacological OCRL inhibition recapitulates defective spreading, persistent filopodia, actin nodules, and impaired marginal microtubule band depolymerization without major loss of GPIb-IX-V or αIIbβ3 receptors, supporting a signaling and cytoskeletal mechanism. Elevated plasma fibrinogen, VWF, and FVIII in LS may partially offset bleeding, though their contribution to the LS phenotype and relationship to vascular/hepatic involvement remains unclear. Similarly, while the OCRL homolog INPP5B is abundant in platelets, its capacity to compensate for OCRL deficiency appears limited and requires further investigation. Recognition of this OCRL-dependent platelet phenotype has important clinical implications, including caution before surgical procedures and usage of medications that may worsen bleeding, highlighting the need for targeted diagnostic approaches and further mechanistic studies to improve the management of individuals with LS.

## Introduction

Lowe syndrome (LS) is a rare X-linked disorder caused by mutations in the oculocerebrorenal syndrome of Lowe gene (OCRL), which encodes the OCRL protein. It affects multiple organs, primarily the eyes, kidneys, and brain. The first manifestations, present at birth, include cataracts and muscular hypotonia, followed later by renal tubular dysfunction, intellectual impairment, arthropathy, and growth delay. Mutations in OCRL also cause Dent-2 disease, which presents with renal dysfunction similar to LS but with milder neurologic and ocular abnormalities (De Matteis et al., 2017). Interestingly, bleeding events have been reported more frequently than expected in individuals with LS, including in the surgical setting (Lasne et al., 2010; Mikhail et al., 2016). Recent studies have identified primary hemostatic defects and platelet function abnormalities as a likely cause of this bleeding tendency. Emerging mechanistic data suggest that loss of OCRL-mediated phosphoinositide turnover perturbs cytoskeletal remodeling at membrane microdomains, thereby impairing platelet adhesion, activation and thrombus formation at sites of vascular injury, providing a plausible molecular basis for the abnormal bleeding phenotype. Here, we review the cellular and molecular basis of the bleeding phenotype observed in LS.

## OCRL

OCRL is a multi-domain protein comprised of an N-terminal pleckstrin homology (PH) domain, a catalytic 5-phosphatase domain, an ASPM-SPD2-Hydin (ASH) domain, and a C-terminal Rho GAP-like domain. ASH domain mediates binding of OCRL to different members of the Rab family of small GTPases localized to secretory and endocytic compartments (De Matteis et al., 2017). The Rho GAP-like domain binds OCRL to the endosomal adaptor proteins (APPL1, IPIP27A) and actin-associated Rho family GTPases Rac and Cdc42 (Faucherre et al., 2005). In addition, through ASH-Rho-GAP-like domains OCRL binds to clathrin heavy chain, AP2, and sorting nexin 9 (SNX9), and thus is involved in clathrin-endocytic trafficking. The OCRL protein is an inositol polyphosphate 5-phosphatase. A major *in vivo* substrate of OCRL is phosphatidylinositol-(4,5)-bisphosphate [PI(4,5)P_2_], catalyzing dephosphorylation at the five-position of the inositol ring, and producing phosphatidylinositol-4-monophosphate (PI4P) (Zhang et al., 1995). *In vitro*, OCRL can also cleave PI(3,4,5)P_3_ as well as soluble inositol(1,4,5)P_3_ (IP_3_). PI(4,5)P_2_ is known to regulate actin dynamics and endocytic trafficking, and serves as a signaling molecule; therefore, OCRL, by hydrolyzing PI(4,5)P_2_, is also involved in regulating those cellular processes.

Within a cell, OCRL is found in several subcellular compartments, including the plasma membrane, clathrin-coated vesicles, early endosomes, lysosomes, and Golgi apparatus (De Matteis et al., 2017). Localization of OCRL protein in various sites of cells could explain the phenotypic heterogeneity of the disease. More than 200 different mutations have been described, with a recent attempt to correlate genotype to phenotype (reviewed in (Brewer, 2025)). It has been perceived that in LS, most mutations localize to exons 13-23, while in Dent-2 disease, which manifests with milder symptoms, most mutations localize to exons 2-12. In addition, mutations that lead to truncated OCRL are more common in LS than in Dent-2 disease, in which mostly non-truncating mutations that change OCRL function occur. Interestingly, thus far bleeding symptoms are not reported in Dent-2 disease.

## Platelets

Platelets are the smallest blood cells with a primary function to regulate hemostasis, but they also participate in pathological processes such as thrombosis. Platelets are anucleated, discoid-shaped cells with granular cytoplasm; they circulate in the bloodstream for 7–10 days, at an approximate concentration of 150–450 × 10^9^/L (Gremmel et al., 2024). After a vascular injury, platelets recognize the damaged vascular wall, become activated, adhere to the exposed extracellular matrix beneath the endothelium, and generate a platelet plug that later develops into a thrombus (van der Meijden and Heemskerk, 2019). Platelets initially adhere to the site of injury through the von Willebrand factor (VWF) receptor (glycoprotein Ib, IX, V; GPIb-IX-V), followed by GPVI receptor binding to collagen. From the discoid resting state, activated platelets spread and increase their surface, with filopodia that will convert into lamellipodia, a process governed by extensive cytoskeletal reorganization (Ghalloussi et al., 2019). Adhered and fully activated platelets provide a procoagulant surface that spatially concentrates and augments the activation of coagulation factors, thereby ensuring efficient stabilization of the platelet plug and cessation of bleeding.

Platelets are formed from megakaryocytes within the bone marrow through two sequential processes: megakaryopoiesis and thrombopoiesis. In megakaryopoiesis, hematopoietic stem cells differentiate into megakaryocytes under the orchestration of thrombopoietin, a key cytokine that drives megakaryocyte differentiation and maturation. Megakaryocyte progenitors expand and develop into enlarged mature megakaryocytes (50–100 µm) that have polyploid nuclei and a complex system of internal membranes (Noetzli et al., 2019). In the next phase, thrombopoiesis, megakaryocytes extend long, branching cytoplasmic protrusions, proplatelets, which ultimately release mature platelets into the bloodstream (Bertovic et al., 2022a). Proplatelet formation relies profoundly on microtubules and the actin cytoskeleton which support extension of cytoplasmic protrusions and branching of proplatelets, respectively.

## Clinical studies reporting hemostatic dysfunction in individuals with Lowe syndrome

Bleeding tendencies have been recognized in LS since early clinical reports. Early studies described hemorrhage in individuals with LS during surgical operations for cataracts, as well as prolonged bleeding after tooth extractions (Curtin et al., 1967; Spierer et al., 1985; Rodrigues Santos et al., 2007). In LS patients, coagulation tests - including prothrombin time, and activated partial thromboplastin time - were found to be within the normal range, indicating intact secondary hemostasis. In contrast, abnormalities in primary hemostasis (platelet function) have been detected in a subset of patients (Lasne et al., 2010; Mikhail et al., 2016; Recker et al., 2015; David et al., 2019; Egot et al., 2021).

**FIGURE 1:**
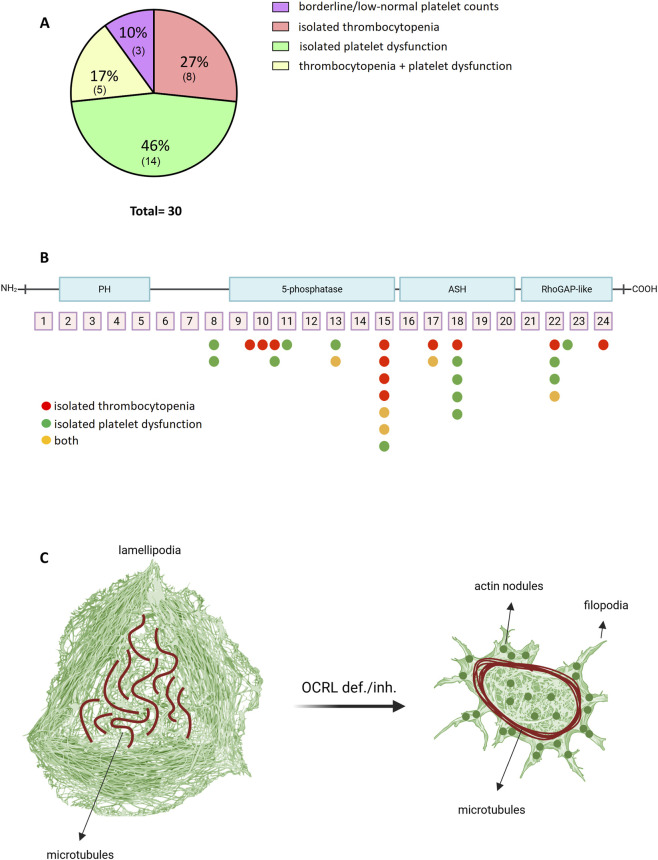
Mutations in the OCRL gene affect platelet function. **(A)**, Frequency of platelet-related characteristics in a group of Lowe syndrome patients with hemostatic phenotype. Within this group, most patients have isolated platelet dysfunction, some have isolated low platelet counts (thrombocytopenia), while only a few have both thrombocytopenia and dysfunctional platelets. **(B)**, Schematic image of OCRL protein domains, and gene exons with mutations (red, green, orange dots) found in Lowe syndrome patients with thrombocytopenia or abnormal platelet function. **(C)**, OCRL regulates the spreading of platelets on fibrinogen and allows lamellipodia formation. If OCRL is deficient (as in LS patients) or pharmacologically inhibited, platelets do not fully spread, exhibit mostly filopodia, actin-rich nodules and preserved microtubular coil. Figures were made in BioRender. NH2, N-term end of Ocrl protein; PH, pleckstrin homology; ASH, ASPM-SPD2-Hydin; COOh, C-term end of Ocrl protein; OCRL def./inh. – OCRL defficient/inhibited.

In Table 1, we summarize OCRL gene mutations, focusing on LS patients with reported decreased platelet number (thrombocytopenia) and/or abnormal platelet function (Lasne et al., 2010; Mikhail et al., 2016; Recker et al., 2015; David et al., 2019; Egot et al., 2021). Among LS patients with hemostatic phenotype, 27% (8 patients) have isolated thrombocytopenia, 10% have borderline/low-normal platelet counts (3 patients), 46% (14 patients) present with isolated platelet dysfunction, and 17% (5 patients) exhibit both thrombocytopenia and abnormal platelet function (Figure 1A). In LS patients with reported hemostatic phenotype, OCRL mutations are distributed from exon eight to the end of the gene. Mutations are clearly enriched in exons 15, 18, and 22, affecting the 5-phosphatase, ASH, and RhoGAP-like domains, respectively, and thereby targeting key regions implicated in phosphoinositide turnover and cytoskeletal regulation (Figure 1B). The more frequent occurrence of mutations in these domains may explain the absence of bleeding reports in Dent-2 disease, which predominantly affects exons 2-12.

**TABLE 1:** Mutations found in Lowe syndrome patients with thrombocytopenia or/and abnormal platelet function. Mutations/exons are shown as reported previously, or determined according to reported transcripts: NM_00276.3 (Recker et al., 2015)*;* NM_00276.4 (Egot et al., 2021).

Patient	Mutation	Exon/Intron	Thrombocytopenia	Abnormal platelet function	Reference
**1**	del OCRL	complete gene deletion	No	Yes	Lasne et al. (2010)
**2**	c.668C>Tc (p.Arg230*)	exon 8	No	Yes	Recker et al. (2015)
**3**	c.646delT p.(Ser216Leufs*34)	exon 8	No	Yes	Egot et al. (2021)
**4**	c.825-2A>G	intron 9	Yes	No	Egot et al. (2021)
**5**	c.836T>C (p.Leu279Pro)	exon 10	Yes	No	Recker et al. (2015)
**6**	c. 940-11G>A	intron 10	No	Yes	Mikhail et al., 2016
**7**	c.940-11G>A	intron 10	Yes	No	Recker et al. (2015)
**8**	c.1000C>T (p.Arg334*)	exon 11	No	Yes	Recker et al. (2015)
**9**	c.1250T>A (p.Val417Asp)	exon 13	Yes	Yes	David et al. (2019)
**10**	c.1262G>A p.(Gly421Glu)	exon 13	No	Yes	Egot et al. (2021)
**11**	c.1262G>A (p.Gly421Glu)	exon 15	No	Yes	Lasne et al. (2010)
**12**	c.1493G>A (p.Cys498Tyr)	exon 15	Yes	Yes	Lasne et al. (2010)
**13**	c.1490G>A (p.Trp497*)	exon 15	Borderline/low-normal	No	Recker et al. (2015)
**14**	c.1493G>A p.(Cys498Tyr)	exon 15	Yes	No	Egot et al. (2021)
**15**	c.1498C>G (p.Arg500Gly)	exon 15	Yes	Yes	Lasne et al. (2010)
**16**	c.1499G>A (p.Arg500Gln)	exon 15	Borderline/low-normal	No	Recker et al. (2015)
**17**	c.1567G>A (p.Asp523Asn)	exon 15	Borderline/low-normal	No	Recker et al. (2015)
**18**	c.1751_1752insG p.(Glu585Glyfs*20)	exon 17	Yes	Yes	Egot et al. (2021)
**19**	c.1822_1834del p.(Ser608Serfs*32)	exon 17	Yes	No	Egot et al. (2021)
**20**	c.1927_1928delGT p.(Val643Asnfs*9)	exon 18	No	Yes	Egot et al. (2021)
**21**	c.1987C>T (p.Arg663*)	exon 18	No	Yes	Lasne et al. (2010)
**22**	c.1987C>T p.(Arg663*)	exon 18	No	Yes	Egot et al. (2021)
**23**	c.2071delA p.(Lys691Lysfs*13)	exon 18	Yes	No	Egot et al. (2021)
**24**	c.2083C>T p.(Arg695*)	exon 18	No	Yes	Egot et al. (2021)
**25**	c.2396C>T p.(Pro799Leu)	exon 22	No	Yes	Egot et al. (2021)
**26**	c.2415C>A p.(Tyr805*)	exon 22	Yes	Yes	Egot et al. (2021)
**27**	c.2428C>T p.(Arg810*)	exon 22	No	Yes	Egot et al. (2021)
**28**	c.2433T>A p.(Cys811*)	exon 22	Yes	No	Egot et al. (2021)
**29**	c.2469+2T>G	intron 22	No	Yes	Lasne et al. (2010)
**30**	c.2581G>C (p.Ala861Pro)	exon 24	Yes	No	Recker et al. (2015)

The first systematic report on platelet dysfunction in LS patients was described by Lasne et al. (2010). The study included six LS patients, of whom four reported a bleeding history during cataract surgery, tooth extraction, or craniostenosis surgery. Clotting tests were normal, although fibrinogen was increased in three cases. Platelet function was assessed using light transmission aggregometry in platelet-rich plasma and platelet-function analyser (PFA), which measures the closure time required for full occlusion of a membrane aperture on collagen-coated cartridges after stimulation with epinephrine or adenosine 5-diphosphate (ADP). This assay depends on platelet adhesion, activation, and aggregation. Interestingly, while aggregation assays were unaffected, platelet closure times were significantly prolonged in all patients. The same research group later described a cohort of 15 LS patients in 2021 (Egot et al., 2021). Almost half (47%) had mild thrombocytopenia (not below 100 × 10^9^/L), and 60% exhibited aberrant platelet function (longer platelet-closure times), while platelet aggregation and of dense granules release were normal (Egot et al., 2021). Interestingly, increased plasma levels of fibrinogen, VWF, and FVIII were present, without evidence of systemic inflammation.

In 2015, Recker et al. conducted a study that characterized the mutational and phenotypic spectrum with 28 LS patients (Recker et al., 2015). Among the phenotypic findings, the authors report haematological abnormalities: six patients had thrombocytopenia or borderline platelet counts (20%), while two additional patients (7%) had a platelet-activation anomaly (Recker et al., 2015).

In a case report, Mikhail et al. described a delayed vitreous haemorrhage after cataract surgery (Mikhail et al., 2016). The authors reported on a 4-week-old male infant with normal platelet counts and coagulation profile, but significantly increased platelet closure times. In another case report, a child exhibited mild thrombocytopenia with abnormal platelet function, and absence of aggregation induced with arachidonic acid, while other aggregation tests were unaffected (David et al., 2019).

Given the observed primary hemostatic dysfunction in LS patients, non-steroidal anti-inflammatory drugs like aspirin or indomethacin could worsen bleeding symptoms (van der Meijden and Heemskerk, 2019). In addition, some anticonvulsant drugs, like carbamazepine or valproic acid, are described to induce haematological abnormalities, including mild thrombocytopenia or anemia (Verrotti et al., 2014; Bartels et al., 2018). Therefore, these medications should be used with caution in LS patients. Further research should determine if cells carrying OCRL mutations are more susceptible to the deleterious effects of these drugs, potentially leading to more pronounced bleeding symptoms.

## OCRL regulation of platelets

In the early study by Lasne et al. (2010), prolonged platelet closure times were explained by a defect in early platelet activation, suggesting inhibition of ROCK, a downstream target of RhoA. In normal platelets, ROCK inhibition increases platelet closure times without effecting platelet aggregation, as observed in LS patients (Lasne et al., 2010). A direct role of OCRL was studied in the later study by the same research group on a cohort of 15 LS patients (Egot et al., 2021)*.* LS platelets showed less efficient thrombus formation under shear conditions on collagen; platelet spreading on a fibrinogen-coated surface was delayed, with most platelets presenting filopodia. In the early phase, clot retraction was impaired, and this was coupled with diminished myosin light chain phosphorylation (MLC), alongside defective Rac1 activation and RhoA overactivation. This impairment of RhoA and Rac1 activation suggest a disturbed balance between contractile and protrusive signaling pathways, and explains why aggregation in suspension assays may remain rather preserved, while spreading and closure under shear are abnormal. In addition, this study showed that megakaryocyte differentiation was preserved in LS patients, while the terminal stage of platelet development, thrombopoiesis, was altered. Megakaryocytes derived from CD34^+^ cells of LS patients, or from cells with silenced OCRL, formed significantly fewer proplatelets that were short and thick with altered actin and tubulin fibers. The peripheral blood of LS patients was enriched with barbell-shaped proplatelets, pointing to premature platelet release. Interestingly, all the abnormalities observed *in vitro* were found both in patients with normal and abnormal bleeding scores, suggesting that OCRL deficiency may be compensated *in vivo* (Egot et al., 2021).

Findings obtained by pharmacological inhibition of OCRL by YU142670 are consistent with the observed phenotype of LS platelets. OCRL-inhibited human platelets do not fully spread on fibrinogen-coated surfaces, and retain numerous actin nodules that are usually replaced by actin stress fibers upon full platelet activation. In addition, OCRL-inhibited platelets fail to progress beyond forming filopodia and preserve the microtubular coil with high levels of acetylated tubulin (Figure 1C) (Bura et al., 2023). Altogether, these genetic and pharmacological findings point to a role for OCRL in regulating both actin and microtubule cytoskeletal dynamics during proplatelet formation and platelet activation.

## OCRL and INPP5B

OCRL shares a significant (51%) sequence identity and domain organization with INPP5B, an enzyme originally identified from human platelets (also called 5-phosphatase II) (Mitchell et al., 1989). However, OCRL was shown to be notably more efficient (10–30 fold) in catalyzing the hydrolysis of PI(4,5)P_2_ than INPP5B (Zhang et al., 1995). Interestingly, while there are many reports of bleeding in LS, there are no reports of mutations in the INPP5B gene linking it to human disease. INPP5B is present in human platelets at approximately double the protein copy number of OCRL (1600 for INPP5B vs. 850 for OCRL (Burkhart et al., 2012)), as well as in mouse platelets (2523 for INPP5B vs. 1039 for OCRL (Zeiler et al., 2014)). However, the higher efficiency of OCRL in catalyzing PI(4,5)P_2_ hydrolysis compared to INPP5B could explain why OCRL deficiency affects platelets in LS (Zhang et al., 1995). On the other hand, higher levels of INPP5B could compensate, to some extent, for the lack of OCRL function in platelets. The pharmacological inhibitor YU142670 has a marked impact on platelet spreading, however it is important to note that this inhibitor acts on both OCRL and INPP5B. OCRL-deficient mice, a humanized mouse strain that expresses human INPP5B while being a double knockout for mouse OCRL and INPP5B, show reduced postnatal growth, low molecular weight proteinuria, aminoaciduria, global muscular dysfunction, and skeletal muscle atrophy [ (B et al., 2011; Festa et al., 2019)]. Although this mouse model does not recapitulate all clinical manifestations of LS and spontaneous bleeding has not been reported, it nonetheless warrants future investigation to elucidate the specific contribution of OCRL to platelet function.

## OCRL in other hematopoietic cells

Beyond platelets, OCRL has been shown to mediate the function of other hematopoietic cells. Interestingly, in *Drosophila melanogaster*, aberrant OCRL function increases the activation of hemocytes, macrophage-like cells. This is paralleled by decreased Rab11-dependent recycling traffic and increased Rab7-dependent late endosomal traffic, leading to augmented immune receptor signaling (Del Signore et al., 2017). However, it is important to stress that *Drosophila* has a single OCRL/INPP5B orthologue. OCRL and INPP5B have been shown to mediate phagocytosis of mammalian macrophages. OCRL is recruited to the phagocytic cup by Rab5, where it removes PI(4,5)P_2_ and promotes phagocytic cup closure (Bohdanowicz et al., 2012; Marion et al., 2012). OCRL was shown to be dispensable for B lymphocytes, while INPP5B modulates B-cell receptor signaling and actin remodeling (Droubi et al., 2022). In addition, microcytosis and anemia have been reported in some LS patients, indicating a possible role of OCRL in erythrocyte biology as well. Renal dysfunction, typically present in this group of patients, could also contribute to anemia; however, this has to be studied further (Lasne et al., 2010; Curtin et al., 1967; Egot et al., 2021).

## Discussion

The accumulated clinical and experimental evidence supports the view that bleeding in LS is mainly caused by a defect in primary hemostasis. OCRL deficiency impairs the early adhesive and cytoskeletal steps required for efficient platelet plug formation at sites of vascular injury. In addition, OCRL contributes to platelet production in addition to platelet function. This dual effect might explain why some patients present with both thrombocytopenia and dysfunctional platelets.

Mechanistically, OCRL regulates phosphoinositide balance, especially PI(4,5)P_2_. OCRL-inhibited platelets have increased levels of intracellular PI(4,5)P_2_ with decreased or unchanged PI4P, depending on the activation status of platelets (Bura et al., 2023; Cheung et al., 2021; Bura and Jurak Begonja, 2021). PI(4,5)P_2_ is crucial for platelet activation, as it regulates cellular processes involved in actin cytoskeleton reorganization, adhesion dynamics, and signaling (Severin et al., 2024; Min and Abrams, 2013). PI(4,5)P_2_ promotes actin polymerization and targets it to the membrane-cytoskeletal interface by mediating the release of barbed-end capping proteins from actin filament ends, such as gelsolin, Arp2/3 complex, and profilin (Hartwig and Barkalow, 1997; Michelson, 2019). During signaling, PI(4,5)P_2_ is a substrate of PLC-δ1, which produces second messengers, IP_3_ and diacylglycerol (DAG) (Michelson, 2019; Gamper and Shapiro, 2007). IP_3_ increases intracellular calcium, which is an essential platelet second messenger that also contributes to the actin reorganization necessary for shape change, degranulation, and inside-out activation of integrin αIIbβ3 (Varga-Szabo et al., 2009). Additionally, PI(4,5)P_2_ is a substrate for class I PI3Ks, producing PI(3,4,5)P_3_ that is transiently increased during platelet activation (Ribes et al., 2020). In addition, OCRL mutations cause increased PI(4,5)P_2_ accumulation at endosomes, leading to aberrant actin filament reassembly that reduces endosomal trafficking. These cellular defects are especially evident and damaging in proximal renal tubules. Platelets from LS patients express normal levels of the most abundant platelet surface receptors: integrin αIIb, GPIbα, and P-selectin (Egot et al., 2021). During platelet activation, GPIbα internalizes and recycles back to the plasma membrane, probably by clathrin-mediated endocytosis (Han et al., 2003; Bertovic et al., 2022b; Eaton et al., 2020). Since several mutations target ASH-Rho-GAP-like domains that mediate clathrin binding, it is possible that OCRL could modulate surface receptor redistribution on platelets, which could delay transduction of signals for full adhesion and activation. However, further studies are needed to confirm this.

Besides PI(4,5)P_2_, OCRL mediates effects on actin cytoskeleton and microtubules through interaction with Rac1, a small GTPase whose active form is reduced in LS platelets (Egot et al., 2021; Severin et al., 2012; Paknikar et al., 2019; van Rahden et al., 2012). Active Rac1 promotes lamellipodia-like actin assembly and can stabilize microtubules. Dysfunction of OCRL disrupts spatial Rac1 signaling, leading to disorganized actin and microtubule architectures. Furthermore, several studies have demonstrated a direct role of PI(4,5)P_2_ in microtubular organization (Lin et al., 2019; Wang et al., 2021). Our study suggests that functional OCRL is necessary for the depolymerization of the microtubular coil during platelet spreading (Figure 1C), and this could also be relevant during proplatelet formation (Bura et al., 2023).

Observed increased levels of fibrinogen, VWF, and FVIII in the plasma of LS patients could reflect compensatory response, but the biological meaning of these findings remains unclear (Lasne et al., 2010; Egot et al., 2021). While platelets (and megakaryocytes) can interact with and internalize fibrinogen *via* endocytosis and release it upon activation, they do not play a major role in regulating plasma fibrinogen concentrations, which are primarily controlled by hepatic synthesis. Elevated plasma VWF and FVIII levels could mirror endothelial activation. Fibrinogen, VWF, and FVIII are primarily cleared through hepatic and reticuloendothelial pathways. LS is not typically associated with overt liver disease; however, given the role of OCRL in intracellular trafficking, indirect hepatic effects cannot be fully ruled out.

The bleeding phenotype appears heterogeneous and incompletely penetrant as some patients show overt perioperative bleeding, whereas others with similar *in vitro* platelet abnormalities have little or no clinically apparent hemorrhage. This implies that OCRL loss creates a biologic vulnerability that may only become clinically relevant under hemostatic stress, such as surgery or trauma. It also suggests that compensatory mechanisms, including residual OCRL activity, INPP5B-mediated phosphoinositide turnover, or interindividual differences in vascular and platelet reserve, may partially buffer the defect *in vivo.* It is possible that some hemorrhaging events have been underrecognized, such as subtle mucocutaneous bleeding or bruising. In addition, the lack of data on female carriers leaves an important gap in risk assessment. Even if carriers are asymptomatic overall, skewed X-inactivation or mosaic OCRL expression could produce measurable platelet abnormalities in some individuals. Therefore, a greater focus on platelet function assessment may help identify LS individuals at increased risk of bleeding, particularly before surgical or invasive procedures. Specialized assays of platelet function (PFA) that measures closure times, are essential to uncover subtle, but functionally significant defects of primary hemostasis. Routine coagulation tests are insufficient to exclude bleeding risk, and platelet number alone may underestimate risk.

Several limitations should be kept in mind when interpreting the current literature. The evidence base is dominated by small cohorts and case reports, and the laboratory methods used across studies are not fully uniform. In the future, larger genotype–phenotype studies are needed to determine which OCRL variants carry the highest bleeding risk and whether specific domains predict platelet dysfunction. Additional work should also evaluate compensatory pathways, especially INPP5B. Humanized animal models and patient-derived megakaryocytes or induced pluripotent stem cell systems could be especially valuable for further clarification on how OCRL deficiency influences platelet function and biogenesis.

## Conclusion

Overall, the evidence indicates that LS includes a genuine hemostatic component rooted in impaired platelet cytoskeletal regulation and abnormal thrombopoiesis. The phenotype is variable, likely modulated by genetic and environmental factors, but it is sufficiently consistent to merit clinical attention. A better mechanistic understanding of OCRL-dependent phosphoinositide signaling may ultimately improve bleeding risk stratification and guide safer management of patients with LS.
